# Prevalence of pelagic dependence among coral reef predators across an atoll seascape

**DOI:** 10.1111/1365-2656.13056

**Published:** 2019-07-25

**Authors:** Christina Skinner, Steven P. Newman, Aileen C. Mill, Jason Newton, Nicholas V. C. Polunin

**Affiliations:** ^1^ School of Natural and Environmental Sciences Newcastle University Newcastle upon Tyne UK; ^2^ Banyan Tree Marine Lab Vabbinfaru Republic of the Maldives; ^3^ NERC Life Sciences Mass Spectrometry Facility Scottish Universities Environmental Research Centre East Kilbride UK

**Keywords:** climate change, connectivity, foraging, plankton, stable isotopes, trophic ecology, trophodynamics

## Abstract

1. Coral reef food webs are complex, vary spatially and remain poorly understood. Certain large predators, notably sharks, are subsidized by pelagic production on outer reef slopes, but how widespread this dependence is across all teleost fishery target species and within atolls is unclear.
2. North Malé Atoll (Maldives) includes oceanic barrier as well as lagoonal reefs. Nine fishery target predators constituting ca. 55% of the local fishery target species biomass at assumed trophic levels 3–5 were selected for analysis. Data were derived from carbon (δ^13^C), nitrogen (δ^15^N) and sulphur (δ^34^S) stable isotopes from predator white dorsal muscle samples, and primary consumer species representing production source end‐members.
3. Three‐source Bayesian stable isotope mixing models showed that uptake of pelagic production extends throughout the atoll, with predatory fishes showing equal planktonic reliance between inner and outer edge reefs. Median plankton contribution was 65%–80% for all groupers and 68%–88% for an emperor, a jack and snappers.
4. Lagoonal and atoll edge predators are equally at risk from anthropogenic and climate‐induced changes, which may impact the linkages they construct, highlighting the need for management plans that transcend the boundaries of this threatened ecosystem.

## Graphical abstract

All sampled fishery target coral reef predators predominantly (65%–88%) relied on planktonic food sources to sustain their biomass in North Malé Atoll, Maldives. Planktonic reliance was equally high inside the atoll lagoon, highlighting the importance of reef–pelagic connectivity and the need for management plans that transcend habitat boundaries.

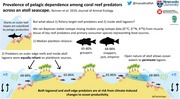

## 1 INTRODUCTION

Until recently, species interactions and nutrient transfer across habitat boundaries and the impact of species declines beyond individual ecosystems were seldom considered (Lundberg & Moberg, 2003). However, ecosystems are now recognized to be linked by flows of organisms and energetic materials (Huxel & McCann, 1998), yet understanding the trophodynamics (the flow of energy) (Lindeman, 1942) of a food web is challenging, particularly for complex marine systems such as coral reefs where spatial variation can be high (Bierwagen, Heupel, Chin, & Simpfendorfer, 2018).

Once thought to be somewhat ecologically closed (Hamner, Colin, & Hamner, 2007; Odum & Odum, 1955), coral reef ecosystems are subject to upwelling and tidal energy, which drive an exchange of plankton, water and nutrients with the ocean (Hamner et al., 2007; Lowe & Falter, 2015). Phytoplankton, a bottom‐up driver of ocean productivity, is often more abundant near islands and atolls (Doty & Oguri, 1956; Gove et al., 2016). Since Darwin (1842), it has been hypothesized that the surrounding ocean provides a major source of nutrition to coral reef communities. Fish on outer reef edges can benefit from this exogenous source (Wyatt, Falter, Lowe, Humphries, & Waite, 2012), but intense feeding by outer reef communities (Genin, Monismith, Reidenhbach, Yahel, & Koseff, 2009) means the energetic material seaward of the reef is different from that in lagoons (Hamner et al., 2007). Furthermore, various hydrodynamic processes are needed to deliver ocean water into the lagoons (Lowe, Falter, Monismith, & Atkinson, 2009), suggesting lagoonal reef fish may not have access to the same resources.

Reef fish communities demonstrate increased reliance on oceanic production seaward of the reef but greater reliance on reef production inshore and into lagoons (Le Bourg et al., 2017; Gajdzik, Parmentier, Sturaro, & Frédérich, 2016; Wyatt, Waite, & Humphries, 2012), indicating that the quantity and quality of food available to inner reef fish varies substantially (Wyatt, Waite, et al., 2012). Variation in nutrient availability and content to the inner and outer reef habitats may lead to spatial differences in reef communities. Indeed, planktivorous fish communities are more abundant with increasing proximity to the ocean (Friedlander, Sandin, DeMartini, & Sala, 2010). Aggregations of these planktivorous fish, the “wall of mouths” (Hamner, Jones, Carleton, Hauri, & Williams, 1988), form on the outer edge of many reefs where they take advantage of increased plankton prey abundances (Wyatt, Lowe, Humphries, & Waite, 2013). The community structure of a coral reef is thus heavily influenced by the adjacent ocean (Garcia, Pelletier, Carpentier, Roman, & Bockel, 2018; Letourneur, 1996; Lowe & Falter, 2015). Oceanic productivity is a key driver of forereef fish biomass (Robinson et al., 2017; Williams et al., 2015), but quantitative estimates of its contribution to lagoonal reef fish biomass are lacking.

Highly mobile reef predators often rely on production sources from outside their primary habitat (McCauley, Young, et al., 2012; Papastamatiou, Meyer, Kosaki, Wallsgrove, & Popp, 2015) and benefit from the aggregations of planktivores (Matley et al., 2018). Some of these predators are partly reliant on oceanic energy fluxes (Frisch, Ireland, & Baker, 2014; Frisch et al., 2016; McCauley, Young, et al., 2012), while others are supported by benthic primary production (Hilting, Currin, & Kosaki, 2013). To date, most of the understanding of these food web relationships comes from studies of reef sharks or from outer forereef slope communities (Frisch et al., 2014, 2016; McCauley, Young, et al., 2012; Papastamatiou, Friedlander, Caselle, & Lowe, 2010). This raises the question of the ubiquity of planktonic reliance in reef fishery target predator communities and whether it extends to those in atoll lagoons.

With climate change, oceanic productivity is projected to decline particularly at low latitudes (Moore et al., 2018) and reef predators could be affected. Yet, the extent of coral reef fishery target species reliance on pelagic production, particularly inside atoll lagoons, is little known. Our study aimed to: (1) determine the level of contribution of planktonic production sources to fishery target reef predator biomass and (2) identify whether this varies between inner lagoonal and outer atoll edge reefs, and among species. In order to address (1), we had to assess fishery target predator species prevalence and biomass across the atoll. We hypothesize that planktonic reliance will be greater along outer edge reefs with reduced reliance in the lagoon where predators will rely more on reef‐based production sources.

## 2 MATERIALS AND METHODS

### 2.1 Study site

The Maldives consists of 16 atolls comprising ocean‐facing edge reefs and enclosed lagoons with patch reefs (Naseer & Hatcher, 2004). The coral reef area is small (8,920 km^2^) (Spalding, Ravilious, & Green, 2001), while the pelagic ocean area within the Exclusive Economic Zone covers ~1 million km^2^ (FAO, 2006). Ocean current flow direction fluctuates with the monsoon. During the Northeast Monsoon, the current flows to the west increasing productivity on the west coast (Sasamal, 2007), while during the Southwest Monsoon, the currents flow to the east increasing primary productivity on the eastern side (Anderson, Adam, & Goes, 2011). Fieldwork was conducted in North Malé Atoll (4°18′34.5″N, 73°25′26.4″E), which is located on the eastern side of the archipelago from January to April 2017 (NE Monsoon). The atoll was divided into two areas: inner atoll/lagoon and outer atoll/edge reef.

### 2.2 Predator community assessments

Underwater visual census (UVC) was used to quantify fishery target predator biomass. Underwater visual census was conducted at 40 sites (20 in each area) covering 50,000 m^2^. These reef fish predators (hereafter “predators”) were mostly piscivore apex predators occupying the upper level of the food chain at assumed trophic positions ≥3. Predators were classified as fishery target species based on current practice in the Maldives from visits to the Malé fish market (C. Skinner, personal observation) and from Sattar, Wood, Islam, and Najeeb (2014). Only forereef habitat was surveyed. At each site, five 50 × 5 m transects were laid haphazardly (minimum 5 m apart) but parallel to the reef at 3–10 m depth. Abundance and size (cm) of all predators were recorded. Predators were characterized based on their behaviour as more mobile or more site‐attached (Brock, 1982). Two observers recorded the predator assemblage; the first laid the transect and recorded mobile species, and the second searched for cryptic, site‐attached species, for example smaller Serranidae. The same observers were used throughout the surveys to prevent observer bias (Willis & Babcock, 2000). Site‐level averages of fish biomass were calculated. All UVC fishery target predator biomass data were calculated using length–weight relationships available on FishBase (http://fishbase.org) with the exception of *Aethaloperca rogaa* where length–weight relationships were taken from Mapleston et al. (2009).

### 2.3 Fish collection

**Figure 1:**
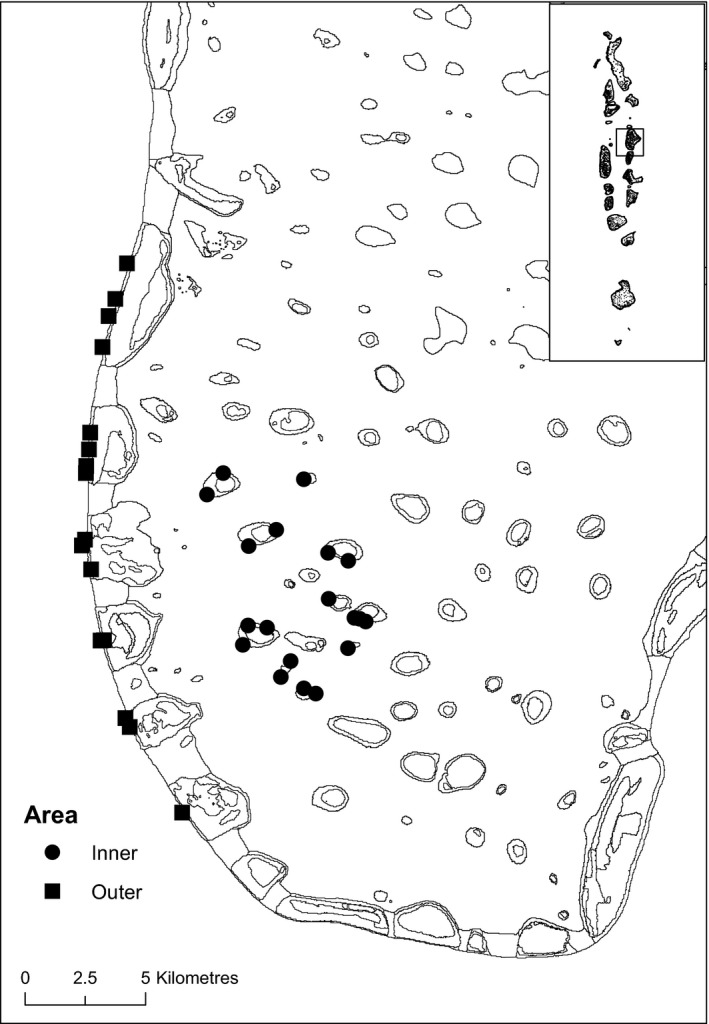
Fish sampling sites in inner lagoonal and outer edge reef areas of North Malé Atoll. Inset is Republic of the Maldives

Fish were collected opportunistically from sites across inner and outer atoll areas for stable isotope analysis (Figure 1). Total length (cm) of each individual was recorded. Samples (1–2 g wet mass) of white muscle tissue from the dorsal musculature adjacent to the dorsal fin were removed. White dorsal muscle was used because it is less variable in δ^13^C and δ^15^N than other tissues (Pinnegar & Polunin, 1999).

Sampled predators were selected based on their prevalence in UVC data, presence in both inner and outer atoll areas, inclusion of species from the dominant fishery target families, and their high trophic position. Nine species belonging to four families were sampled: groupers (Serranidae: *A. rogaa*, redmouth grouper, *n* = 22; *Anyperodon leucogrammicus*, slender grouper, *n* = 20; *Cephalopholis argus*, peacock grouper, *n* = 21; *Cephalopholis miniata*, coral hind, *n* = 21), snappers (Lutjanidae: *Aphareus furca*, jobfish, *n* = 8; *Lutjanus bohar*, red snapper, *n* = 13; *Lutjanus gibbus*, humpback snapper, *n* = 22), emperors (Lethrinidae: *Lethrinus obsoletus*, orange‐striped emperor, *n* = 5) and jacks (Carangidae: *Caranx melampygus*, bluefin trevally, *n* = 16). Predators were captured using rod and reel, handlines and pole spears. Where possible (e.g. when caught using handlines), sampling was non‐lethal using 4‐mm biopsy punches (Henderson, Stevens, & Lee, 2016).

Different primary producers vary in ratios of δ^13^C and δ^34^S, with distinct values typically associated with benthic versus planktonic algae (France, 1995) and marine habitat types, respectively. Food web analysis typically uses δ^13^C, but δ^34^S helps to discriminate between different production pathways as there is often greater variability in mean S isotopic value of sources compared to C or N (Connolly, Guest, Melville, & Oakes, 2004). Here, food sources were characterized through sampling a range of primary consumers that feed on specific food groups. Primary consumers can be used as a reference baseline for elucidating trophic positions in the food web with greater certainty than those of primary producers as they incorporate variability and have slower tissue turnover times (Cabana & Rasmussen, 1996; Vander Zanden & Rasmussen, 1999). Primary consumers were chosen based on dietary information from the published literature. Six energy pathways were represented: (a) benthic algae (*Acanthurus leucosternon*, powderblue surgeonfish, 6 inner, 11 outer (Robertson, Polunin, & Leighton, 1979)); (b) hard corals (*Chaetodon meyeri*, scrawled butterflyfish, 5 inner, 11 outer (Sano, 1989)); (c) detritus (*Pearsonothuria graeffei*, blackspotted sea cucumber, 7 inner, 8 outer (Purcell, Samyn, & Conand, 2012)); (d) diurnal plankton (*Caesio xanthonota*, yellowback fusilier, 11 inner, 2 outer (Bellwood, 1988); *Caesio varilineata*, variable‐lined fusilier, 12 inner (Bellwood, 1988); *Decapterus macarellus*, mackerel scad, 20 inner (Smith‐Vaniz, 1995); *Pterocaesio pisang*, banana fusilier, 12 inner (Bellwood, 1988)); (e) nocturnal plankton (*Myripristis violacea*, lattice soldierfish, 11 inner, 6 outer (Hobson, 1991)); and (f) diel vertically migrating (DVM) plankton (*Uroteuthis duvaucelii*, Indian Ocean squid, 7 outer (Islam, Hajisamae, Pradit, Perngmak, & Paul, 2018)). Although an effort was made to consistently sample primary consumers, *U. duvaucelii* does not feed directly on DVM plankton but on small crustaceans and fishes (e.g. bottom‐dwelling sea robins, *Trigla* sp. (Islam et al., 2018)). However, they reside at depths of 30–170 m and feed primarily at night when they migrate to shallower waters, so they were considered a suitably representative proxy for DVM plankton. Several species of planktivores were sampled to control for the greater variability occurring across plankton communities. Primary consumer species were collected using pole spears or from Malé fish market.

### 2.4 Stable isotope analysis

Tissue samples were oven‐dried at 50°C for 24 hr and then freeze‐dried before grinding to a homogenous powder using a pestle and mortar. Approximately 2.5 mg was weighed into 3 × 5 mm tin capsules and analysed for δ^13^C, δ^15^N and δ^34^S using a PyroCube elemental analyser (Elementar) interfaced with an Elementar VisION IRMS at the NERC Life Sciences Mass Spectrometry Facility, East Kilbride, UK. Stable isotope ratios are reported using the delta (δ) notation with measured values expressed in per mil (‰), where δ is [(R_sample_/R_standard_) – 1] x 1000 and R is the ratio of heavy to light isotope (e.g. ^13^C/^12^C). Four international reference materials were used at the start and end of each C/N/S run and three internal reference materials every ten samples to ensure accuracy and correct for drift (Table S1). Analytical precision (*SD*) for international standard USGS40 was 0.1 and 0.2 for δ^13^C and δ^15^N, respectively, and for IAEA‐S1, IAEA‐S2 and IAEA‐S3, it was 0.2, 0.6 and 1.5 for δ^34^S, respectively. Analytical precision (*SD*) for internal reference materials M2, MSAG2 and SAAG2 was 3.2, 0.1 and 0.1 for δ^13^C, 3.2, 0.2 and 0.1 for δ^15^N and 1.7, 0.5 and 0.5 for δ^34^S, respectively. Accuracy between runs was assessed using a randomly spaced study‐specific reference (mature *A. leucogrammicus,* TL = 41.4 cm). Analytical precision (*SD*) was 0.1 for δ^13^C, 0.3 for δ^15^N and 0.7 for δ^34^S.

Carbon stable isotope data were lipid‐corrected arithmetically when the C:N ratio of the muscle tissue was >3.7 using the mass balance equation of Sweeting, Polunin, and Jennings (2006):

(1) $$\delta_{\text{protein}}=\frac{\left(\delta_{\text{sample}}\times {\text{C:N}}_{\text{sample}}\right)+\left(7\times \left({\text{C:N}}_{\text{sample}}-{\text{C:N}}_{\text{protein}}\right)\right)}{{\text{C:N}}_{\text{sample}}}$$

Lipid corrections were applied to only 20 predator samples (*A. rogaa*, *C. melampygus*, *C. miniata*, *L. gibbus*) and 12 primary consumer samples (exclusively *P. graeffei*). Mean (*SD*) differences in δ^13^C values after correction were 1.2 (1.0) and 1.0 (0.9), respectively.

### 2.5 Statistical analyses

All analyses were carried out using r Statistical Software version 3.5.1 (R Development Core Team, 2010) and RStudio version 1.1.383 (RStudio Team, 2012).

Predator abundance data were square‐root‐transformed, and a Bray–Curtis similarity matrix was made. Using the “vegan” r package (Oksanen et al., 2018), differences in predator abundances between areas were assessed using a perMANOVA with 999 permutations. Species contributing to these differences were identified using SIMPER analysis.

Bayesian stable isotope mixing models were run using the r package “mixsiar” (Stock & Semmens, 2016a) to ascertain the predators' principal food sources. Each model was run using three tracers (δ^13^C, δ^15^N and δ^34^S) with area (inner/outer) as a fixed factor and species as a random factor. The error term Residual * Process was selected as residual error incorporates potential variation involving consumers, for example differences in metabolic rate or digestibility, while process error incorporates variation related to the sampling process (e.g. *L. bohar* *n* = 1 sample size in the outer atoll) (Stock & Semmens, 2016b). Models were run using the “very long” MCMC parameters. Model convergence was assessed using the trace plots and the Gelman–Rubin and Geweke diagnostic tests.

Source contribution estimates can be highly uncertain when there are too many sources (Ward, Semmens, Phillips, Moore, & Bouwes, 2011). For the best separation of source contributions, it is recommended that sources are combined prior to analysis based on biological knowledge and similar isotopic values (a priori) or, where source isotope values differ, that estimated proportional contributions are combined following analysis (a posteriori) (Phillips, Newsome, & Gregg, 2005). Here, sources were represented by the sampled primary consumer species. Sources were combined a priori when (a) they were the same species or they represented the same food source and (b) there were no significant differences in their isotope values. δ^13^C, δ^15^N and δ^34^S values of the (a) primary consumer species sampled in both inner and outer atoll areas and (b) the four diurnal planktivore species were compared using ANOVAs or, where data did not conform to normality or homoscedasticity, Kruskal–Wallis tests. In some cases, source isotope values may be statistically different even when they have similar isotope values. When this occurred, the mean isotope values of each source were calculated. If the difference in the mean values was small (~1‰), they were combined a priori (Phillips et al., 2014).

A mean isotopic value and standard deviation was determined for each group to represent the different sources in the mixing models. Several sources were then combined a posteriori. This approach allows each individual source to be included in the running of the model while combining sources after may provide a narrower combined distribution with greater biological relevance (Phillips et al., 2014, 2005). Differences in the δ^13^C, δ^15^N and δ^34^S values of the reef‐based group and planktonic source group were assessed using a Kruskal–Wallis test.

Trophic discrimination factors (TDF, Δ) vary depending on many factors, and inappropriate TDF can result in misinterpretations. Because of this, four models were run using different TDFs. Trophic discrimination factors were chosen as they were calculated based on white muscle tissue from upper trophic level predatory fish in marine environments, and when plotted, the consumer data were inside the polygon made by the source data. **Model 1** used in situ values field‐estimated from Palmyra Atoll for Δδ^13^C and Δδ^15^N: +1.2 (*SD* ± 1.9) and +2.1 (*SD* ± 2.8), respectively (McCauley, Young, et al., 2012). Little published information is available on Δδ^34^S, but it is thought to be around 0‰ (Peterson & Fry, 1987). In a feeding study of European sea bass (*Dicentrarchus labrax*), Barnes and Jennings (2007) calculated Δδ^34^S to be −0.53 (*SD* ± 0.04), but it ranged from −1.59 to +0.26. Therefore, Δδ^34^S *SD* was increased to 1.0 to incorporate this variability and provide additional model parameter space. **Model 2** used the Δδ^13^C = +0.4 (*SD* ± 0.2) and Δδ^15^N = +2.3 (*SD* ± 0.3) for aquatic environments from McCutchan, Lewis, Kendall, and McGrath (2003) and the same Δδ^34^S as model 1. **Model 3** used values from Vander Zanden, Casselman, and Rasmussen (1999) for carnivores, Δδ^13^C = +0.9 (*SD* ± 1.0) and Δδ^15^N = +3.2 (*SD* ± 0.4) and the same Δδ^34^S as model 1. **Model 4** used Δδ^13^C + 1.2 (*SD* ± 1.9) and Δδ^15^N + 2.1 (*SD* ± 2.8) from McCauley, Young, et al. (2012) and a Δδ^34^S of +1.9 (*SD* ± 0.51) for aquatic environments from McCutchan et al. (2003); however, the model did not converge and the consumer source data were outside the source mixing polygon.

The predictive accuracy of the different models was compared using the r package “loo” (Vehtari, Gabry, Yao, & Gelman, 2018) (Table S5). Leave‐one‐out‐cross‐validation (LOO) assesses Bayesian model prediction accuracy (Vehtari, Gelman, & Gabry, 2017). The model with the lowest LOO value and the highest Akaike weight was **model 1,** which is presented in the results (Stock et al., 2018). However, the same patterns remained with the different TDFs (Figure S2). Although median values of plankton contributions vary, the fundamental concepts are consistent: (a) planktonic reliance is a significant contributor to fishery target reef predator biomass, and (b) this reliance extends into inner atoll areas.

## 3 RESULTS

**Figure 2:**
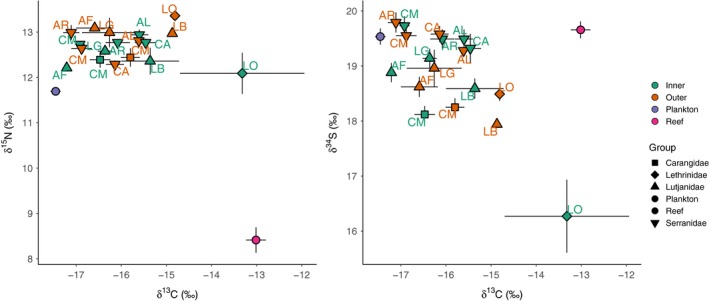
Mean isotope values (±*SE*) of (a) δ^13^C and δ^15^N and (b) δ^13^C and δ^34^S of combined “reef” and “plankton” primary consumer groups (circles) sampled to represent different end‐members and reef predators sampled in inner atoll and outer atoll. Predators in group order: CM = *Caranx melampygus*, LO = *Lethrinus obsoletus*, AF = *Aphareus furca*, LB = *Lutjanus bohar*, LG = *Lutjanus gibbus*, AL = *Anyperodon leucogrammicus*, AR = *Aethaloperca rogaa*, CA = *Cephalopholis argus*, CM = *Cephalopholis miniata*

Of 30 fishery target species in five families recorded by UVC, nine in four families were sampled for stable isotope analysis in both inner and outer atoll areas (Figure 1). The average predator biomass (±*SD*) across the study sites was 127.9 ± 107.9 kg/ha (100.3 ± 78.7 kg/ha inner; 155.5 ± 126.9 kg/ha outer). The sampled species constituted 58% of the predator assemblage (60% or 60.6 ± 39.8 kg/ha inner; 55% or 84.8 ± 66.2 kg/ha outer). The predator assemblages differed between atoll areas (perMANOVA, 999 permutations, *p* < .01), but only one of the sampled predators, *A. leucogrammicus*, contributed significantly to this (SIMPER, *p* < .01) and it was more abundant in the inner atoll. Mean δ^13^C values (±*SE*) ranged from −17.1 ± 0.2 to −13.3 ± 1.4 (*A. rogaa*, outer atoll, to *L. obsoletus*, inner atoll), δ^15^N from 12.1 ± 0.4 to 13.4 ± 0.1 (*L. obsoletus*, inner atoll, to *L. obsoletus*, outer atoll) and δ^34^S from 16.2 ± 0.7 to 19.8 ± 0.2 (*L. obsoletus*, inner atoll, to *A. rogaa*, outer atoll; Figure 2a,b; Table S2).

There were significant differences in isotopic data of three primary consumer species between atoll areas: *C. meyeri* (hard coral) (ANOVA, δ^15^N: *F*_1,14_ = 6.5, *p* < .05); *M. violacea* (nocturnal plankton) (Kruskal–Wallis, δ^15^N: $\chi_{1,15}^{2}$ = 4.5, *p* < .05); and *P. graeffei* (detritus) (ANOVA, δ^15^N: *F*_1,13_ = 4.7, *p* < .05; δ^13^C: *F*_1,13_ = 14.9, *p* < .05; and δ^34^S: *F*_1,13_ = 8.0, *p* < .05; Table S3). These differences were small (~1‰) so these sources were combined a priori (Table S4; Figure S3). There were no significant differences between the areas for the remaining primary consumer species (ANOVA or Kruskal–Wallis, *p* > .05). δ^15^N and δ^34^S values did not differ significantly among diurnal planktivores *C. varilineata* (mean ± *SE*: δ^15^N 11.5 ± 0.1; δ^34^S 19.1 ± 0.2), *C.* *xanthonota* (mean ± *SE*: δ^15^N 11.6 ± 0.3; δ^34^S 18.9 ± 0.3), *D. macarellus* (mean ± *SE*: δ^15^N 11.7 ± 0.2; δ^34^S 19.2 ± 0.2) or *P. pisang* (mean ± *SE*: δ^15^N 11.5 ± 0.1; δ^34^S 18.9 ± 0.3) (ANOVA, *p* > .05) but δ^13^C values did (Kruskal–Wallis, δ^13^C: $\chi_{1,53}^{2}$ = 30.1, *p* < .01; Table S3). As the differences in δ^13^C values were small (~1‰), these species were combined into one food source group (hereafter “Diurnal planktivores” [Table S4; Figure S3]).

A posteriori*,* the food sources (represented by the primary consumers) benthic algae, coral and detritus were combined into one “reef” food source group (hereafter “reef” sources), while nocturnal plankton, diurnal plankton and DVM plankton were combined into one “plankton” food source group. The δ^13^C and δ^15^N values of the reef‐based and planktonic‐based primary consumer end‐members were highly significant different (δ^13^C: Kruskal–Wallis, $\chi_{1}^{2}$ = 80.6, *p* < .01; and δ^15^N: $\chi_{1}^{2}$ = 67.9, *p* < .01, respectively; Figure 2a; Figure S3a). Planktonic primary consumers all had more negative δ^13^C signatures, while reef‐based primary consumers had less negative δ^13^C, indicating benthic energy pathways (Figure 2a; Figure S3a). The reef‐based and plankton‐based δ^34^S scarcely differed ($\chi_{1}^{2}$ = 1.9, *p* > .05; Figure 2b; Figure S3b).

**Figure 3:**
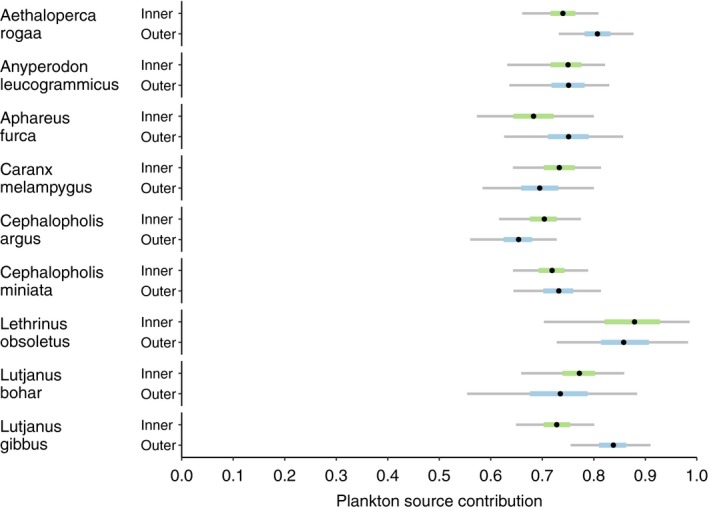
Results of Bayesian mixing model with applied trophic discrimination factors, which determined the plankton source contribution to the nine reef predators in both inner and outer atolls. Thick bars represent credible intervals 25%–75%, while thin bars represent 2.5%–97.5%. Black dots represent the medians (50%)

Mixing models indicated that all nine predators were predominantly (65%–88%) sustained by planktonic food sources in both inner and outer atolls (Figure 3; Table S6). Median plankton reliance was highest for *L. obsoletus* in the inner atoll (88%) and lowest for *C. argus* in the outer atoll (65%). Differences in reliance between areas for each species were small and ranged from 0.1% to 11%.

Groupers in both areas derived 65%–80% of their biomass from planktonic food sources, while reef sources contributed only 20%–35%. Between areas, contributions did not vary by more than 6%. *A. rogaa* had higher median planktonic reliance in the outer atoll (80% outer, 74% inner), while *C. argus* had higher median reliance in the inner atoll (70% inner, 65% outer). Median values for *A. leucogrammicus* and *C. miniata* were equal in both atoll areas (75% both; 72% inner, 73% outer, respectively). Credible intervals were similar for all four grouper species.

The median planktonic reliance range of snappers, emperor and jack was 68%–88%. Both *A. furca* and *L. gibbus* had higher median planktonic reliance in the outer atoll than in the inner atoll (75% outer, 68% inner; 84% outer, 73% inner, respectively), whereas *L. bohar* had a slightly higher median reliance on plankton in the inner atoll (77% inner, 73% outer). *Lethrinus obsoletus* had almost equal median planktonic reliance in both areas (86% inner, 88% outer). Of all the predators, *L. gibbus* had the biggest difference in median reliance between atoll areas (11%). Credible intervals for *L. gibbus* were small, while those for *L. obsoletus* and outer atoll *L. bohar* were largest. *Caranx melampygus* had greater median plankton reliance in the inner atoll (73% inner, 69% outer), and credible intervals were similar to the groupers. There was substantial overlap in the proportional planktonic contribution estimates of all the predators in both areas.

## 4 DISCUSSION

Planktonic production was the primary contributor to reef fishery target predator biomass regardless of proximity to the open ocean. These results add to growing evidence (Frisch et al., 2014; McCauley, Young, et al., 2012; Wyatt, Waite, et al., 2012) that oceanic productivity is crucial for sustaining the biomass of many coral reef fish communities; this planktonic dependence is prevalent among the main predators, and in the present case, it clearly extends to lagoonal reefs. These identified linkages are not necessarily ubiquitous to coral reef systems, however. In the Northwestern Hawaiian Islands, over 90% of apex predator biomass was sustained by benthic primary production (Hilting et al., 2013), highlighting how trophodynamics may vary substantially spatially, even among similar systems.

Plankton was the predominant contributor to biomass for all of the predators sampled. These predator families have a known reliance on nekton (Kulbicki et al., 2005). Given the high diversity and biomass of planktivores on Maldivian reefs (McClanahan, 2011; Moritz et al., 2017) and the relatively small home ranges of the sampled predators (Karkarey, Alcoverro, Kumar, & Arthur, 2017; Sattar, 2009; Sluka & Reichenbach, 1995), we hypothesize that they link adjacent pelagic and reef ecosystems by primarily feeding on planktivorous prey. Cross‐system linkages, similar to those found here, are increasingly being documented. In the Solomon Islands, the piscivorous coral trout *Plectropomus leopardus* is sustained by feeding on planktivorous fish (Greenwood, Sweeting, & Polunin, 2010). In Palmyra Atoll, a circuitous ecological interaction chain was discovered where δ^15^N from seabird guano over preferred native forests led to increased abundances and biomasses of zooplankton in adjacent waters (McCauley, Desalles, et al., 2012). Similarly in the Chagos Archipelago, on islands free of invasive rats, seabird densities were higher, leading to increased N deposition from offshore foraging, increasing reef fish community biomass (Graham et al., 2018). These semi‐pristine environments provide an opportunity to identify these linkages and determine how anthropogenic and climate‐induced impacts may affect them.

The high degree of planktonic dependence in predators on lagoonal reefs suggests that planktonic resources are readily available across both atoll areas. Similarly, coral host and POM δ^13^C and δ^15^N did not differ between inner and outer reefs in the central Maldives (Radice et al., 2019). Although there is little published information on the internal hydrodynamics of North Malé Atoll, these results suggest that lagoonal waters are providing planktonic subsidies to inner reef communities, but it is unclear whether they come from outside the atoll or from internal hydrodynamic characteristics of the lagoon. In Palmyra Atoll, inner and outer regions are well connected by a range of hydrodynamic processes (Rogers, Monismith, Fringer, Koweek, & Dunbar, 2017). Mixing inside lagoons arises from wave forcing over reef crests and vortices, generated from the wake of flow separation from currents hitting the atoll, help to redistribute water to different regions (Rogers et al., 2017). Internal waves and surface downwelling are also key distributors of particulate‐rich waters (Williams et al., 2018). However, these findings are in contrast to Ningaloo, Western Australia, and Mo'orea, French Polynesia, where δ^13^C and fatty acids of reef fish (Wyatt, Waite, et al., 2012) and the δ^13^C, δ^15^N and δ^34^S of damselfish (Gajdzik et al., 2016), respectively, showed a gradient in oceanic reliance, decreasing into the lagoons. While the lagoons of both Ningaloo and Mo'orea are fairly constricted, North Malé lagoon is substantially more open. We hypothesize that the porosity and open nature of the atoll render lagoonal conditions similar to the open ocean. Future work to identify how nutrients circulate and enter into the lagoons would allow this transfer of energetic materials to be better understood.

The Maldives experiences substantial monsoonal fluctuations in productivity (Radice et al., 2019). As such, timing and location of sampling may influence the degree of planktonic reliance. Here, sampling occurred on the eastern side of the archipelago during the NE season, that is when productivity is supposedly lower. Additionally, due to the double chain nature of the Maldivian archipelago, the outer atoll sites surveyed were adjacent to other atolls, rather than to the pelagic ocean. Despite this, planktonic production was the predominant contributor to predator biomass. This further supports the hypothesis that the porosity of the atoll allows oceanic resources to permeate, and as a result, Maldivian coral reefs are heavily influenced by the open ocean regardless of location and season.

Although interspecific differences in plankton reliance were apparent, median values were high and similar between areas for each species. *Lethrinus obsoletus* had the highest plankton reliance in both areas (~87%). Emperors often forage over soft bottom habitats where they feed on prey such as molluscs and crustaceans (Kulbicki et al., 2005). Many of these may reflect planktonic signatures as they feed on plankton via filter feeding (Jørgensen, 1966) or in the water column at night (McMahon, Thorrold, Houghton, & Berumen, 2016). *Lethrinus nebulosus* on Ningaloo reef slopes also relies on oceanic productivity, but in the lagoon, it is sustained by reef‐based production (Wyatt, Waite, et al., 2012), perhaps further indication that variation in lagoonal hydrodynamics may influence food web structure. *Lethrinus obsoletus* also had larger credible intervals. While these were likely confounded by small sample size (*n* = inner 3, outer 2), they may also reflect variability in the range of isotope values. Inner atoll *L. obsoletus* isotope values covered a broader range (range δ^13^C: 4.8‰; δ^15^N: 1.5‰; and δ^34^S: 2.3‰) than in the outer atoll (range δ^13^C: 0.2‰; δ^15^N: 0.2‰; and δ^34^S: 0.3‰), indicating that individuals in the lagoon have a larger isotopic niche than their forereef conspecifics. Niche width depends on the diversity of resources available (Araújo, Bolnick, & Layman, 2011). The greater availability of soft bottom habitat in the lagoon may offer a wider range of prey.

Outer atoll *C. argus* had the lowest plankton reliance (65%). *Cephalopholis argus* are generalist predators that prey on a wide range of reef‐associated fish (Dierking, Williams, & Walsh, 2011; Harmelin‐Vivien & Bouchon, 1976), so greater benthic reliance is probable. However, the median value of 65% indicates that two thirds of their biomass is supported by planktonic subsidies, higher than expected given previous dietary studies. *Cephalopholis argus* can exhibit foraging plasticity (Karkarey et al., 2017) and readily switch prey groups (Shpigel & Fishelson, 1989). As such, they may be opportunistically foraging on planktivores, a dominant component of Maldivian reefs (McClanahan, 2011). Similarly on the Great Barrier Reef, *Plectropomus* species primarily foraged on the most abundant prey families, Pomacentridae and Caesionidae, indicating that they were opportunistic generalists (Matley et al., 2018). The ability of *C. argus* to switch prey may confer a competitive advantage, allowing them to survive fluctuations in prey communities resulting from environmental change (Karkarey et al., 2017).

The predator assemblage differed significantly between areas, but only one of the sampled predators, *A. leucogrammicus*, contributed significantly. Evidently, the sampled predators constitute an important part of the assemblage and are key components of the biomass in each area. Furthermore, irrespective of minor differences in median plankton reliance, all the predators had substantially overlapping credible intervals. Even *L. gibbus*, where median plankton reliance differed most between areas (inner 75%, outer 86%), had credible intervals, which overlapped considerably with the other species. This may indicate a degree of interspecific competition, raising the question of how they partition resources. Further investigation of their dietary niches is the recommended next step for this work.

Underwater visual census has been the main method for assessing reef fish populations, but it can undersample more mobile species (White, Simpfendorfer, Tobin, & Heupel, 2013; Willis & Babcock, 2000). To account for such shortcomings, 50‐m transects (a total of 1,250 m^2^ surveyed reef at each site from five transects) were used to increase the likelihood of encountering mobile predators (McCauley, Young, et al., 2012), while baited underwater video deployed in the same areas (C. Skinner et al. unpublished data) identified the same fish species as the most prevalent.

Multiple primary consumers were sampled to attempt to comprehensively characterize the potential production sources at the base of the reef food web. Planktivorous primary consumers may differ isotopically due to differing preferences among the diverse plankton taxa, so several planktivorous primary consumers were sampled. Although the primary consumers representing “reef” and “plankton” separated out isotopically, future studies would benefit from validating each primary consumer by characterizing the food source they represent and including multiple primary consumers to represent each end‐member, for example bristle‐toothed surgeonfish *Ctenochaetus striatus* as an alternate detritivore (Tebbett, Goatley, Huertas, Mihalitsis, & Bellwood, 2018) or chevron butterflyfish *Chaetodon trifascialis* as an alternate corallivore (McMahon, Berumen, & Thorrold, 2012).

Reef predators are important fishery targets, providing food security and ecosystem services to millions globally (Cinner et al., 2018). Herein, they are found to play an important ecological role linking adjacent ecosystems (McCauley, Young, et al., 2012). Projected declines in oceanic productivity, particularly at low latitudes (Bopp et al., 2013; Moore et al., 2018), may severely impact these Maldivian predators and the linkages they construct. Marine protected areas (MPAs) are widely used in coral reef conservation, but reliance of many reef fish on non‐reef production sources suggests the protection MPAs offer is susceptible to climate‐induced changes. To adequately address these potential impacts on coral reef food webs, managers need to move towards management plans that transcend the boundaries of these threatened ecosystems.

## AUTHORS' CONTRIBUTIONS

All persons who qualify under authorship criteria are listed as authors, and all take responsibility for the content of the article. C.S., S.P.N. and N.V.C.P. formulated the ideas; C.S., S.P.N., A.C.M. and N.V.C.P. developed methodology; C.S. conducted fieldwork; C.S. and J.N. processed samples; A.C.M. and C.S. analysed the data; C.S. wrote the manuscript; and all authors edited the manuscript.

## Data Availability

Data used in this study are available from the Dryad Digital Repository: https://doi.org/10.5061/dryad.7jj53hb (Skinner, Newman, Mill, Newton, & Polunin, 2019).
