# Supplementary material for: Prevalence of pelagic dependence among coral reef predators across an atoll seascape
Source: J Anim Ecol. 2019 Jul 25;88(10):1564–74. doi: 10.1111/1365-2656.13056 (PMC6852557; doi:10.1111/1365-2656.13056)
Supplement: Supplementary file 1 [file JANE-88-1564-s001.docx]

Online Resource: Electronic Supplemental Material

Prevalence of pelagic dependence among coral reef predators across an atoll seascape.

Christina Skinner^1,^*, Steven P Newman^1,2^, Aileen C Mill^1^, Jason Newton^3^, Nicholas VC Polunin^1^

1 = School of Natural and Environmental Sciences, Newcastle University, Newcastle upon Tyne, NE1 7RU, UK

2 = Banyan Tree Marine Lab, Vabbinfaru, Republic of the Maldives

3 = NERC Life Sciences Mass Spectrometry Facility, Scottish Universities Environmental Research Centre, Rankine Avenue, East Kilbride G75 0QF, UK

* corresponding author = [c.e.skinner@ncl.ac.uk](mailto:c.e.skinner@ncl.ac.uk); +44 (0) 191 208 5607

**Table S1**. Accepted and measured values ± SD of the international, internal and study-specific reference materials used during the stable isotope analyses. International standards were USGS40 (glutamic acid) for δ^13^C and δ^15^N ([Qi et al. 2003](#_ENREF_5)) and silver sulfide standards IAEA- S1, S2 and S3 for δ^34^S ([Coplen and Krouse 1998](#_ENREF_2)). The internal reference materials were MSAG2 (a solution of methanesulfonamide and gelatin), M2 (a solution of methionine, gelatin, glycine and ^15^N-enriched alanine) and SAAG2 (a solution of sulfanilamide, gelatin and ^13^C-enriched alanine). The selected internal references cover a large range of isotopic composition and are in solution form, so easily dispensed by syringe.


 **Table S2**. Mean (± S.E) body length (mm) and stable isotope (δ^13^C, δ^15^N, δ^34^S) values (‰) for each reef predator species sampled in both inner and outer atoll.

**Table S3**. Summary of ANOVAs comparing δ^13^C, δ^15^N and δ^34^S values between inner and outer atoll areas and between species. † = data did not conform to normality so a non-parametric Kruskal-Wallis test was used instead. Significance is denoted by ‘*’. Following these statistical tests *Caesio varilineata,* *Caesio xanthanota, Decapterus macarellus* and *Pterocaesio pisang* were combined in to one group named “Diurnal planktivores”.

**Table S4**. Mean (± S.E) stable isotope (δ^13^C, δ^15^N, δ^34^S) values (‰) for each primary consumer species sampled in both inner and outer atoll. Bold indicates statistical differences in isotope values of the samples species were found between areas using ANOVA or Kruskal-Wallis tests. When differences in the mean were small (~1‰), samples from each area were combined for each group.

**Table S5**. Comparison of mixing models fit using MixSIAR on the reef predator diet data using four different trophic discrimination factors. dLOOic = difference in LOOic between each model and the model with the lowest LOOic ([Stock et al. 2018](#_ENREF_6)). The model with the lowest LOOic and the highest weight was presented in the results. Model 1 had a 55% probability of being the best model while model 2 had a 45% probability of being the best model suggesting both are equally good. * indicates the model did not converge.

**Table S6**. Credible intervals of plankton source contribution from a three-source (δ^13^C, δ^15^N and δ^34^S) Bayesian stable isotope mixing model run to ascertain likely food source contributions for nine reef predator species.

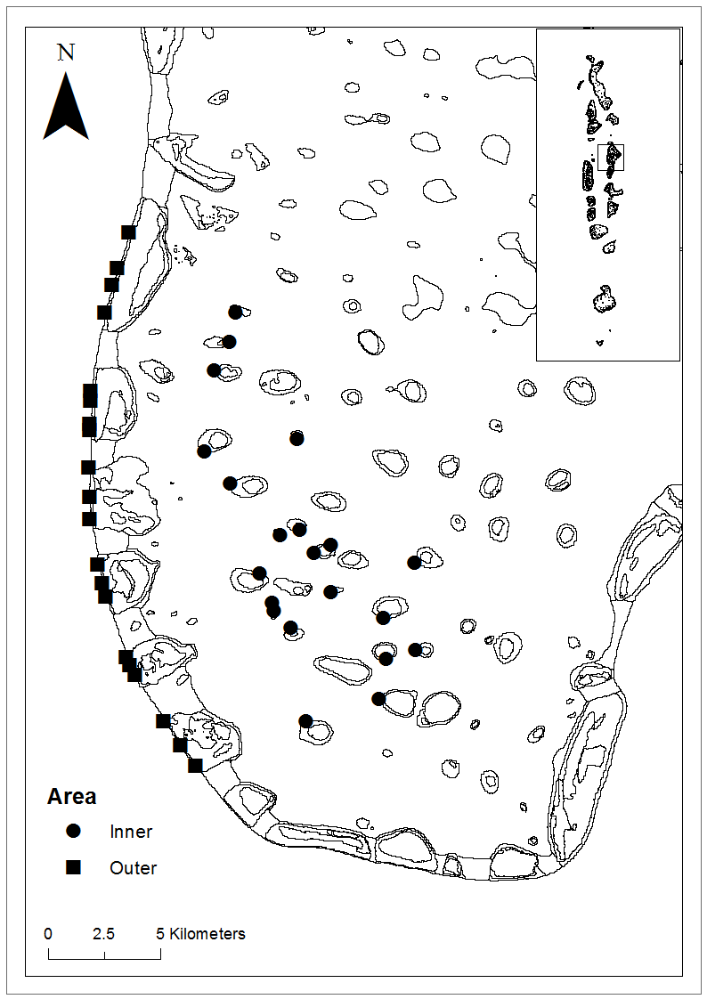


**Figure S1**. UVC sampling sites in inner lagoonal and outer edge reef areas of North Malé atoll.

**Trophic Discrimination Factors (TDF)**

TDF vary depending on many factors and the use of inappropriate TDF can result in misinterpretations. It is therefore advisable to try different TDFs when running Bayesian stable isotope mixing models.

Three additional Bayesian stable isotope mixing models were run using different Δδ^13^C and Δδ^15^N. **Model 2** used the Δδ^13^C = +0.4 (SD ± 0.2) and Δδ^15^N = +2.3 (SD ± 0.3) for aquatic environments from [McCutchan Jr et al. (2003)](#_ENREF_4) and the same Δδ^34^S = -0.53 (SD ± 1.00) from [Barnes and Jennings (2007)](#_ENREF_1) (Fig. S2a). **Model 3** used values from ([Vander Zanden et al. 1999](#_ENREF_7)) for carnivores, Δδ^13^C = 0.9 (SD ± 1.0) and Δδ^15^N = 3.2 (SD ± 0.4) and the same Δδ^34^S = -0.53 (SD ± 1.00) from [Barnes and Jennings (2007)](#_ENREF_1) (Fig. S2b). **Model 4** was run using Δδ^13^C 1.2 (SD ± 1.9) and Δδ^15^N 2.1 (SD ± 2.8) from [McCauley et al. (2012)](#_ENREF_3) and a Δδ^34^S of 1.9 (SD ± 0.51) for aquatic environents from [McCutchan Jr et al. (2003)](#_ENREF_4) however the model did not converge and the consumer source data were outside the source mixing polygon. With these different TDFs (Fig. S2a; Fig. S2b) the same patterns remain. Although median values of plankton contributions vary the fundamental concepts are consistent between both sets of models; 1) planktonic reliance is a significant contributor to fishery target reef predator biomass and 2) this reliance extends in to inner atoll areas.

| (a)  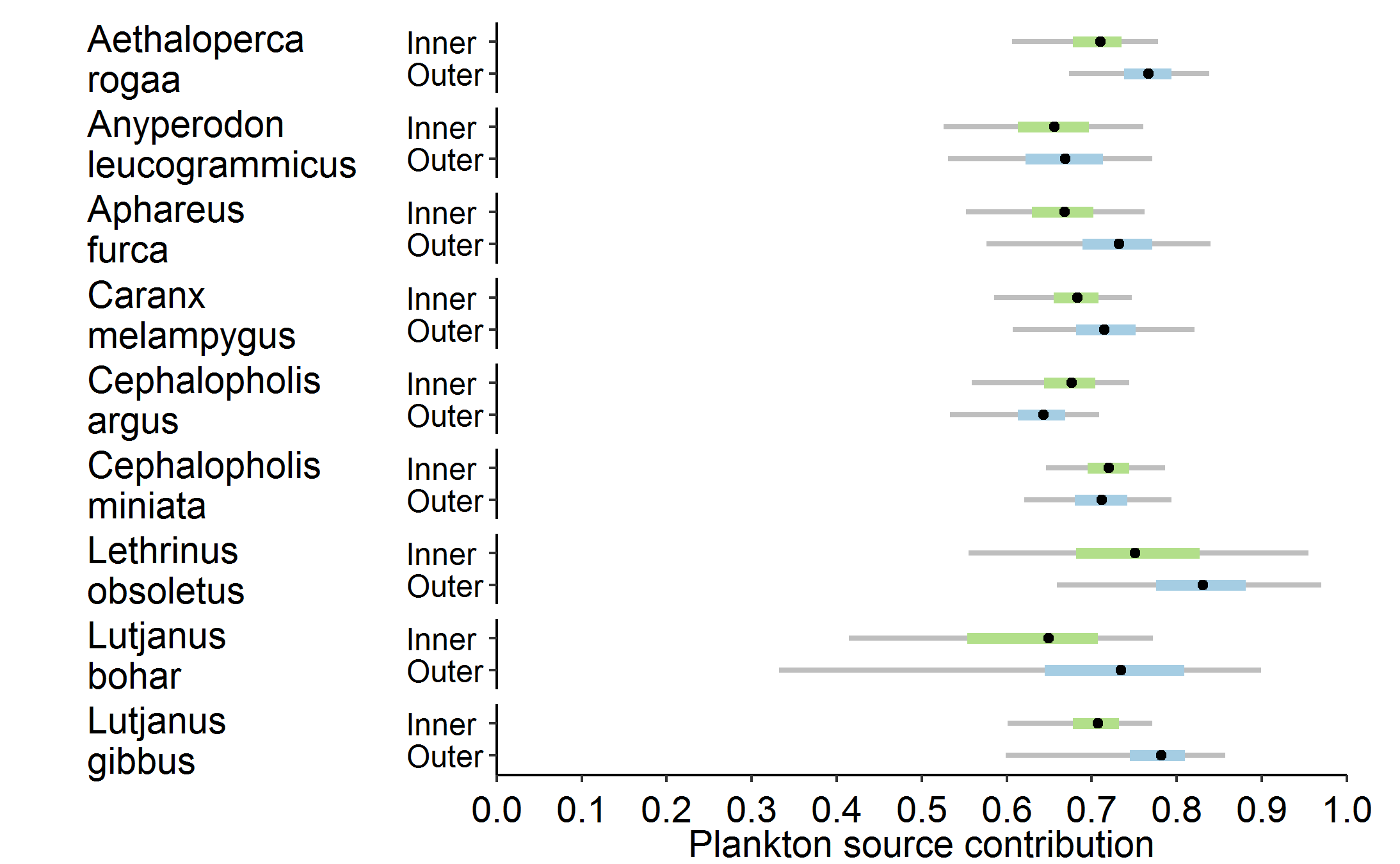 |
| --- |
| (b)  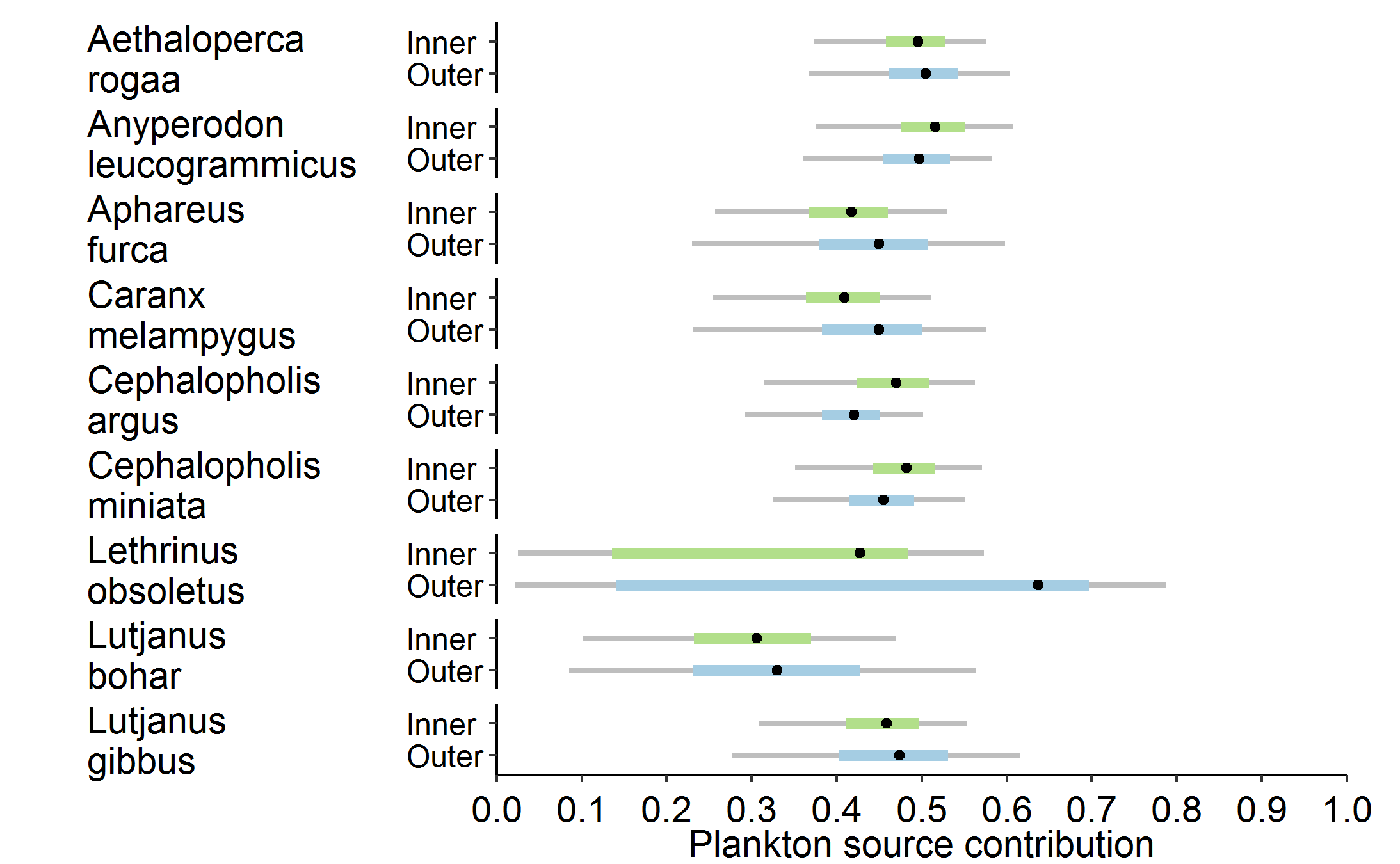 |

**Figure S2**. Results of Bayesian mixing models testing different trophic discrimination factors (TDFs) which determined the principal food source contributions to the nine reef predator species sampled in both inner and outer atoll areas. TDFs used for each model were a) Δδ^13^C = +0.4 (SD ± 0.2) and Δδ^15^N = +2.3 (SD ± 0.3) for aquatic environments ([McCutchan Jr et al. 2003](#_ENREF_4)) and Δδ^34^S = -0.53 (SD ± 1.00) ([Barnes and Jennings 2007](#_ENREF_1)), and b) Δδ^13^C = 0.9 (SD ± 1.0) and Δδ^15^N = 3.2 (SD ± 0.4) for carnivores ([Vander Zanden et al. 1999](#_ENREF_7)) and Δδ^34^S = -0.53 (SD ± 1.00) ([Barnes and Jennings 2007](#_ENREF_1)). Thick bars represent credible intervals 25-75% while thin bars represent 2.5-97.5%. Black dots represent the medians (50%).

**
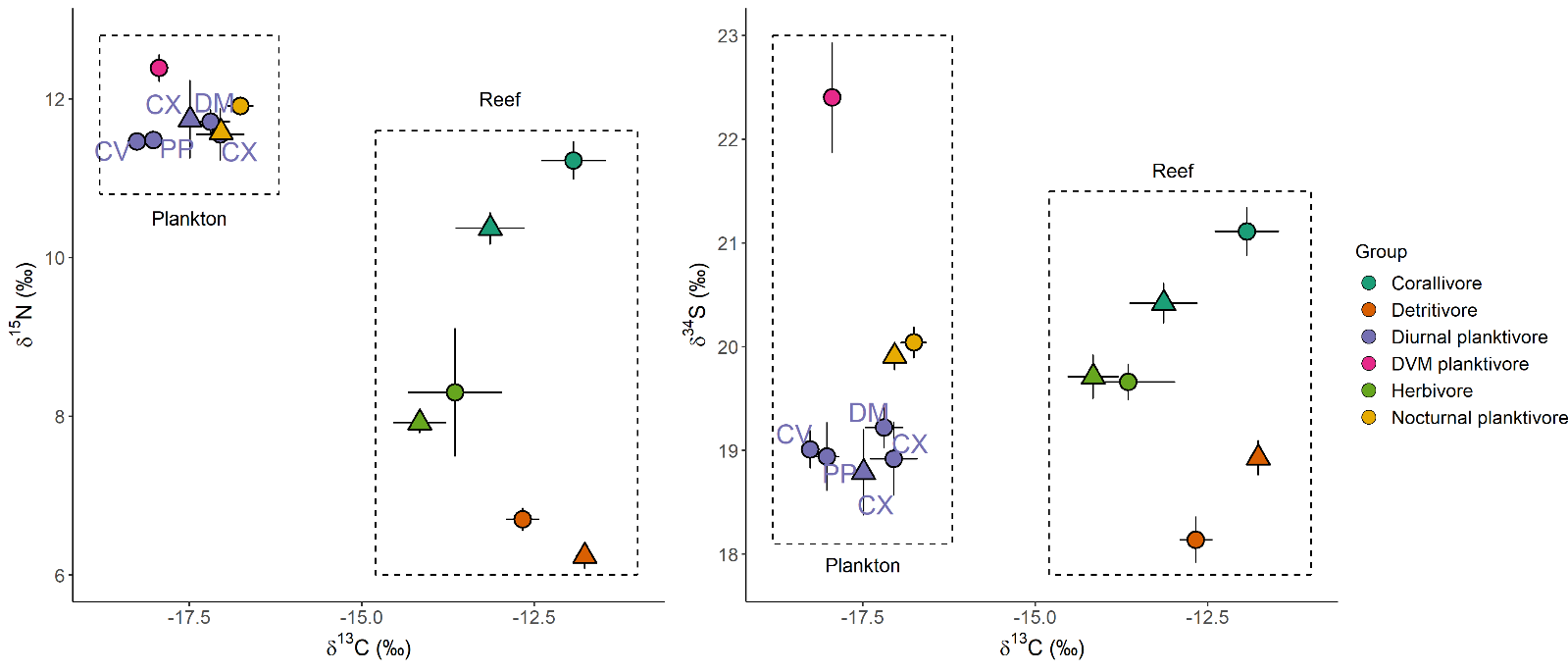
**

**Figure S3**. Mean isotope values (± SE) of a) δ^13^C and δ^15^N and b) δ^13^C and δ^34^S of all primary consumers sampled to represent different end-members in both inner (●) and outer (▲) atoll before they were combined *a priori*. Boxes show *a posteriori* groupings. Four species of diurnal planktivores were sampled: CV: *Caesio varilineata*, CX: *Caesio xanthanota*, DM: *Decapterus macarellus* and PP: *Pterocaesio pisang*.

Barnes C, Jennings S (2007) Effect of temperature, ration, body size and age on sulphur isotope fractionation in fish. Rapid communications in mass spectrometry : RCM 21:1461-1467

Coplen TB, Krouse HR (1998) Sulphur isotope data consistency improved. Nature 392:32

McCauley DJ, Young HS, Dunbar RB, Estes JA, Semmens BX, Michel F (2012) Assessing the effects of large mobile predators on ecosystem connectivity. Ecological Applications 22:1711-1717

McCutchan Jr J, Lewis Jr W, Kendall C, McGrath C (2003) Variation in trophic shift for stable isotope ratios of carbon, nitrogen, and sulfur102:378-390

Qi H, Coplen TB, Geilmann H, Brand WA, Böhlke JK (2003) Two new organic reference materials for δ13C and δ15N measurements and a new value for the δ13C of NBS 22 oil. Rapid Communications in Mass Spectrometry 17:2483-2487

Stock BC, Jackson AL, Ward EJ, Parnell AC, Phillips DL, Semmens BX (2018) Analyzing mixing systems using a new generation of Bayesian tracer mixing models. PeerJ 6:e5096

Vander Zanden JM, Casselman JM, Rasmussen JB (1999) Stable isotope evidence for the food web consequences of species invasions in lakes Nature 401:464-467
